# A hierarchical processing unit for multi-component behavior in the avian brain

**DOI:** 10.1016/j.isci.2021.103195

**Published:** 2021-09-30

**Authors:** Noemi Rook, John Michael Tuff, Julian Packheiser, Onur Güntürkün, Christian Beste

**Affiliations:** 1 Department of Biopsychology, Institute of Cognitive Neuroscience, Faculty of Psychology, Ruhr-University Bochum, Universitätsstraße 150, 44801 Bochum, Germany; 2 Max Planck School of Cognition, Stephanstrasse 1a, 04103 Leipzig, Germany; 3 Cognitive Neurophysiology, Department of Child and Adolescent Psychiatry, Faculty of Medicine, TU Dresden, 01307 Dresden, Germany; 4 University Neuropsychology Center, Faculty of Medicine, TU Dresden, 01307 Dresden, Germany

**Keywords:** Biological sciences, Neuroscience, Behavioral neuroscience, Cognitive neuroscience

## Abstract

Multi-component behavior is a form of goal-directed behavior that depends on the ability to execute various responses in a precise temporal order. Even though this function is vital for any species, little is known about how non-mammalian species accomplish such behavior and what the underlying neural mechanisms are. We show that humans and a non-mammalian species (pigeons) perform equally well in multi-component behavior and provide a validated experimental approach useful for cross-species comparisons. Applying molecular imaging methods, we identified brain regions most important for the examined behavioral dynamics in pigeons. Especially activity in the nidopallium intermedium medialis pars laterale (NIML) was specific to multi-component behavior since only activity in NIML was predictive for behavioral efficiency. The data suggest that NIML is important for hierarchical processing during goal-directed behavior and shares functional characteristics with the human inferior frontal gyrus in multi-component behavior.

## Highlights

• Pigeons and humans perform equally well in the STOP-CHANGE paradigm
• We identified relevant brain regions for the examined behavioral dynamics in pigeons
• ZENK expression in NIML was predictive for behavioral efficiency
• This study provides a validated experimental approach for cross-species comparisons

## Graphical abstract

**Figure:**
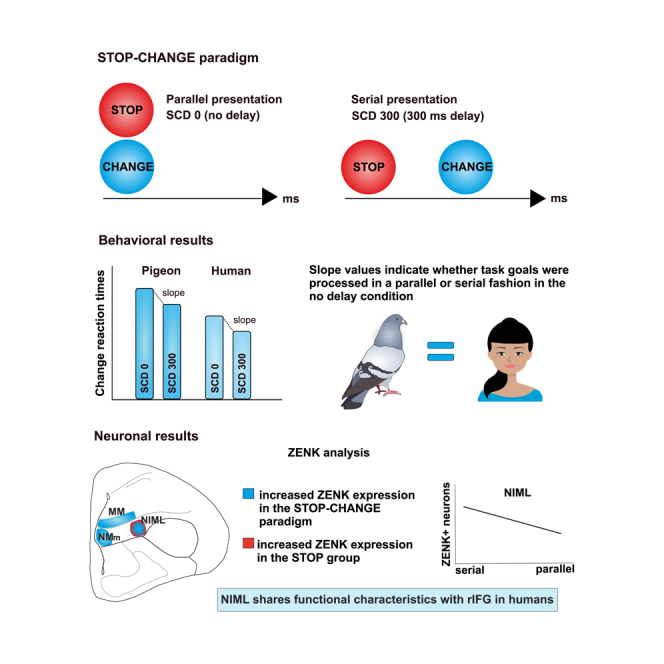

## Introduction

Humans and other animals are confronted with various response options in everyday life situations that need to be organized to achieve a goal. Especially in dynamic environments, agents have to respond in real-time with multiple response possibilities competing for limited resources at the same time. Both humans and animals can use different strategies to cope with the demands of such multi-component behavior. While some individuals process task goals in a “serial” fashion, others seem to apply a rather “parallel” processing strategy (Verbruggen et al., 2008; Verbruggen and Logan, 2009). It has been found that a parallel processing mode results in less efficient multi-component behavior compared with a more serial goal activation (Dippel and Beste, 2015; Verbruggen et al., 2008). This is the case as response selection capacities are restricted, and the simultaneous activation/parallel processing of two task goals likely overstrains capacity limits, which creates interference between task goals (Meyer and Kieras, 1997; Mückschel et al., 2014; Stock et al., 2014; Verbruggen et al., 2008; Verbruggen and Logan, 2009). In contrast to this, a serial goal activation does not overstrain these capacity limits leading to more efficient multi-component behavior (Dippel and Beste, 2015; Verbruggen et al., 2008).

Multi-component behavior can be tested with a STOP-CHANGE paradigm, which is an extended version of the well-known STOP signal paradigm (Dippel and Beste, 2015; Mückschel et al., 2014; Stock et al., 2014). In both paradigms, an already initiated GO response needs to be stopped in some trials as indicated by a STOP signal. However, the STOP-CHANGE paradigm includes a third signal that indicates a CHANGE response. Thus, the STOP-CHANGE paradigm has a greater complexity as it requires subjects to perform multiple actions (GO, STOP, and CHANGE) to achieve their goals. Importantly, within STOP-CHANGE paradigms, the processing strategy can be investigated by introducing variations in the delay between STOP and CHANGE stimulus presentation. When there is no delay, one has the choice to process the STOP and CHANGE task goals at the same time or to process these goals step-by-step. However, when the delay between the STOP and CHANGE signals is longer than the time needed to respond to the STOP signal, one is forced to process STOP and CHANGE stimuli in a serial manner. The processing mode of multi-component behavior can be determined by comparing the reaction times to the CHANGE stimuli of both conditions (Verbruggen et al., 2008) (see STAR Methods section for more details).

In humans, frontal and parietal areas belong to the multiple demand system, which is associated with a variety of cognitive tasks including multi-component behavior (Beste et al., 2019; Beste and Saft, 2015; Dippel and Beste, 2015; Duncan, 2010; Gohil et al., 2016; Mückschel et al., 2014, 2017; Ness and Beste, 2013; Stock et al., 2014, 2015). Of these areas, the right inferior frontal gyrus (rIFG) is of particular relevance for the hierarchical organization of actions (Binkofski and Buccino, 2006; Duncan, 2010; Fiebach and Schubotz, 2006; Koechlin and Jubault, 2006), organizing individual components of action sequences (Charron and Koechlin, 2010; Clerget et al., 2011), and the processing strategy of multi-component behavior (Dippel and Beste, 2015). Moreover, the basal ganglia have been linked to this form of goal-directed behavior based on their role in action selection and action chunking (Beste and Saft, 2015; Ness and Beste, 2013; Stock et al., 2014). However, as methodological possibilities to study the neural circuits and molecular mechanisms of multitasking are limited in humans, appropriate animal models are desperately needed in this area of research. Pigeons are a classical animal model of learning and behavior with outstanding task engagement (Güntürkün et al., 2017), and recent evidence has shown that pigeons are able to cope with the demands of multi-component behavior (Rook et al., 2020). Moreover, despite their partly different forebrain organization the circuitry of the avian and mammalian pallium is highly similar suggesting that pigeons might also be an appropriate neuronal model (Stacho et al., 2020). Most importantly however, studying diverse animal species offers the possibility to identify critical variables that jointly occur in differently organized brains and thus possibly define core neural constituents of cognition/action selection (Strausfeld and Hirth, 2013). Similarly, comparative research can also help to investigate whether neurocomputational models reflect biological necessities or are just one of several possible solutions.

In pigeons, the avian nidopallium caudolaterale (NCL), which is regarded as a functional equivalent to the mammalian prefrontal cortex (PFC), as well as the homologous striatum are active during the STOP-CHANGE paradigm (Rook et al., 2020). However, recent fMRI data suggests that the action control system in birds might also include areas of the medial nidopallium/mesopallium (MNM) (Behroozi et al., 2020). Areas within this region have moreover been associated with fast sensorimotor learning and sequential behavior in chicks and pigeons (Helduser and Güntürkün, 2012; Horn, 2004). In songbirds, one component of MNM is involved in song learning and sequencing, whereas other areas within this complex are activated during limb and body movements (Feenders et al., 2008). In pigeons, functional subdivisions within MNM have not yet been defined, but the nidopallium intermedium medialis pars laterale (NIML) seems to be of particular relevance for serial order behavior (Helduser and Güntürkün, 2012) and might thus be crucial for the processing strategy of multi-component behavior. In order to investigate the functional role of MNM in pigeons during the STOP-CHANGE paradigm, different groups of pigeons performed either simple movement execution (GO group), response inhibition (STOP signal group), or multi-component behavior (STOP-CHANGE group). MNM was subdivided into the medial mesopallium (MM), the nidopallium mediale pars medialis (NMm) and NIML for a subsequent immediate-early gene expression analysis to investigate potential functional differences. To ensure that the STOP-CHANGE paradigm in pigeons investigates similar behavioral and cognitive processes as the STOP-CHANGE paradigm in humans, the behavioral outcome of both species in their paradigms was directly compared with each other.

## Results

### Efficiencies of pigeons and humans in the STOP-CHANGE paradigm

**Figure 1:**
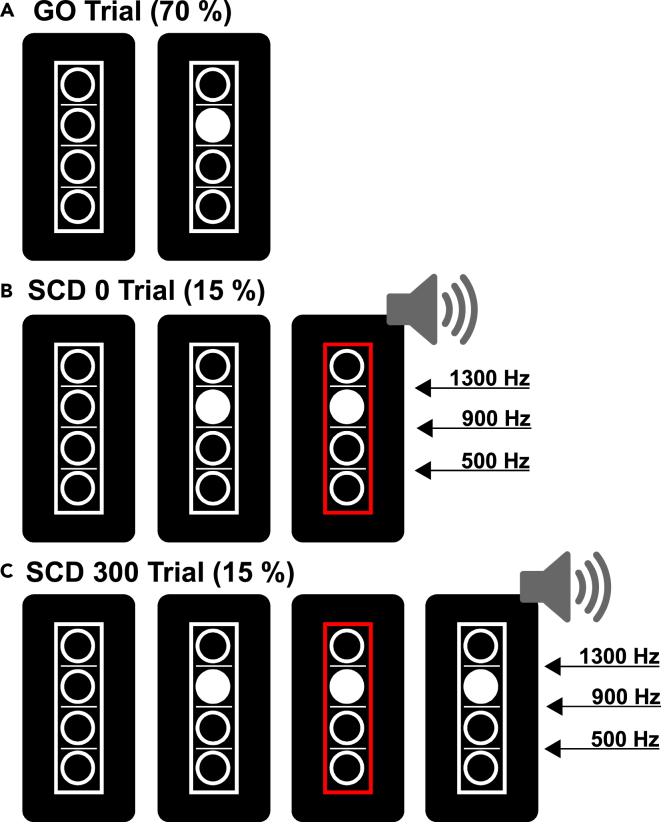
Illustration of the paradigm that was applied to study multi-component behavior in humans (A) Illustration of a typical GO trial. Participants were asked to estimate whether the filled white circle was above or beneath the reference line in the middle and had to press a corresponding key on a 4 button response pad. (B and C) Illustration of typical STOP-CHANGE trials. In those trials the GO response needed to be inhibited and a tone indicated which reference line should be used instead of the middle line. Participants had to make their response based on the corresponding key on a keypad. (B) Illustration of the SCD 0 condition where the CHANGE signal (tone) and the STOP signal (red square) were presented simultaneously. (C) Illustration of the SCD 300 condition where the STOP signal (red square) was presented 300 ms prior to the CHANGE signal (tone). The delay between GO onset and the appearance of the red STOP signal (SSD) was varied based on a staircase procedure. The delay between the red STOP signal and the white CHANGE stimulus (STOP-CHANGE delay, SCD) was in 50% of the cases 0 ms (SCD 0 condition) and in the other 50% 300 ms (SCD 300 condition).

**Figure 2:**
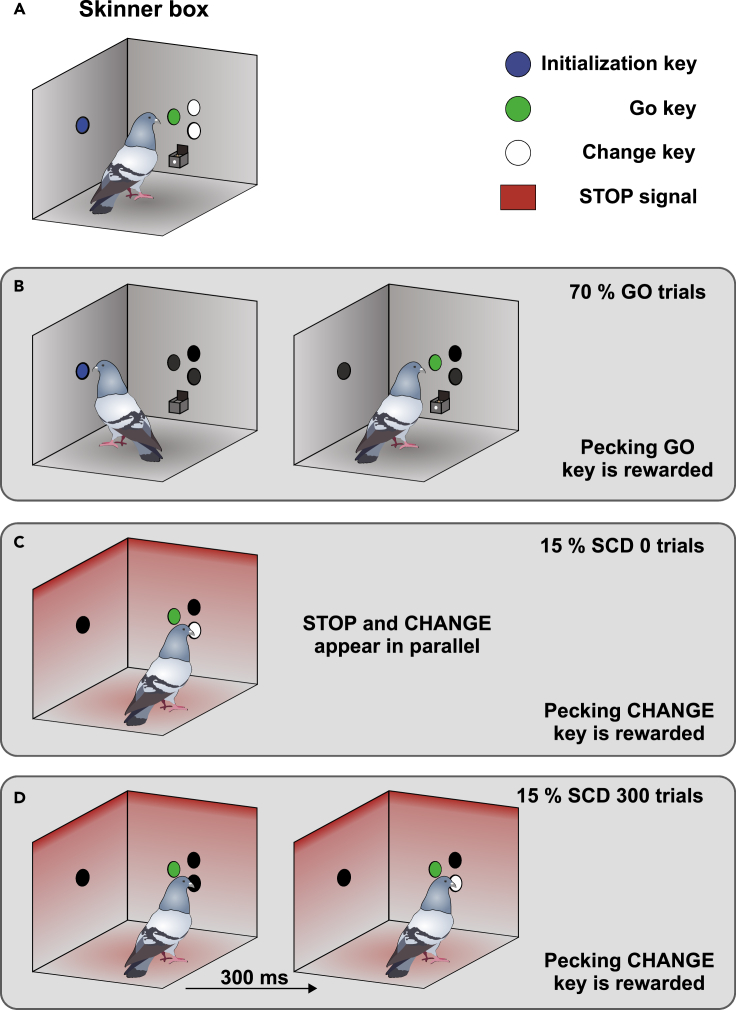
Illustration of the STOP-CHANGE paradigm in pigeons (A) Schematic illustration of the Skinner box that was used in this paradigm. On the left side, the blue initialization key is located, whereas the green GO key and the two white CHANGE keys are located on the rear wall above the feeder. (B) Schematic illustration of a typical GO trial that occurred in 70% of cases and in which pecking the green GO stimulus was the rewarded action that finished the trial. (C) Schematic illustration of the SCD 0 condition, which occurred in 15% of cases. In these trials, pecking the white CHANGE key was rewarded and finished the trial. In this condition, STOP and CHANGE appear simultaneously. (D) Schematic illustration of the SCD 300 condition, which also occurred in 15% of all cases. In these trials, pecking the white CHANGE key was also the rewarded action and finished the trial. However, in the SCD 300 condition, a 300 ms time delay was employed between the STOP and CHANGE stimuli. The CHANGE signal appeared in a randomized order either in the top or bottom location. Pecking the green GO stimulus once the red STOP signal appeared was counted as a mistake. In all SC trials the delay between GO onset and the appearance of the red STOP signal (SSD) was varied based on a staircase procedure.

First of all, we determined whether pigeons are a suitable behavioral animal model showing comparable performances to humans in the STOP-CHANGE paradigm. Therefore, pigeons (n = 20) and humans (n = 20) were both tested in highly comparable versions of the STOP-CHANGE paradigm (Figures 1 and 2). Both the pigeon and human paradigm consisted of 70% GO trials in which a GO response was the correct action (Figures 1A, 2A, and 2B). The remaining 30% of the trials were so called STOP-CHANGE trials, where a STOP signal indicated that the GO response needed to be inhibited and a CHANGE signal indicated that a CHANGE response needed to be executed instead. The appearance of the STOP signal (STOP signal delay, SSD) was adjusted by a “staircase procedure” (Verbruggen et al., 2008). When the participant was successful in inhibiting their GO response and also correctly reacted to the CHANGE key, 50 ms were added to the SSD in the next trial (to make stopping more difficult). When participants were unsuccessful in one of those actions, 50 ms were subtracted from the SSD in the next trial (to make stopping easier). This procedure was applied to end up with a probability of 50% successful STOP-CHANGE trials (SC trials). Moreover, these SC trials were subdivided into 15% SCD 0 and 15% SCD 300 trials for both species. In SCD 0 trials, the STOP and CHANGE signals were presented simultaneously (Figures 1B and 2C), whereas in SCD 300 trials the STOP and CHANGE signals were separated by a 300 ms time delay (Figures 1C and 2D). The efficiency of multi-component behavior can then be determined by contrasting the reaction times of the two experimental conditions SCD 0 and SCD 300 (slope values, for more details see STAR Methods section).

**Figure 3:**
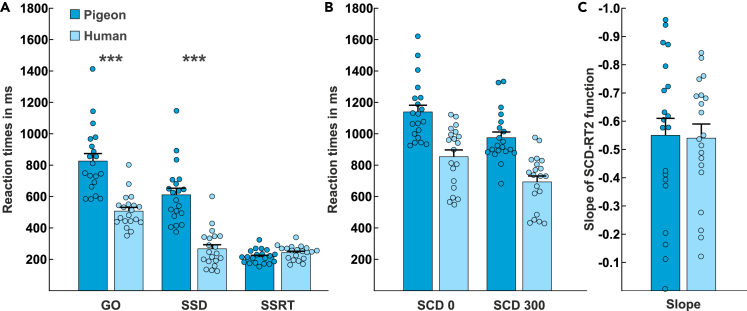
Comparison of reaction times of pigeons and humans in the STOP-CHANGE paradigm (A) Mean reaction times in the GO condition, SSDs and SSRTs of pigeons (dark blue) and humans (light blue). (B) Reaction times of the SCD 0 and SCD 300 conditions of pigeons (dark blue) and humans (light blue). (C) Slope of the SCD-RT2 function that indicates the processing mode of multi-component behavior for pigeons (dark blue) and humans (light blue). We did not find species differences between humans (n = 20) and pigeons (n = 20) in SSRTs or in the efficiency of multi-component behavior (slope value). SC RTs, GO RTs and SSD length were significantly different between humans (n = 20) and pigeons (n = 20). Error bars present the standard error of the mean (SEM). Dots represent the reaction times/slopes of the individuals. GO RTs, SSDs, SSRTs and slope values of pigeons and humans were compared with Bonferroni corrected independent samples t-tests. Reaction times in the SCD 0 and SCD 300 conditions of pigeons and humans were compared with a repeated measures ANOVA. ∗∗∗p < 0.001.

GO reaction times (GO RTs), SSDs, stop signal reaction times (SSRTs) and slope values of pigeons and humans were compared with independent samples t-tests. To correct for multiple comparisons and avoid alpha error accumulation, the alpha significance levels were Bonferroni adjusted and set to 0.0125. We found that pigeons had significantly longer GO RTs than humans (GO_pigeon_: 826 ms ± 48 SEM, GO_human_: 507 ms ± 24 SEM, t_(27.943)_ = 6.011, p < 0.001*,* Figure 3A), which also resulted in a significantly longer SSD for pigeons compared with humans (SSD_pigeon_: 611 ms ± 42 SEM, SSD_human_: 267 ms ± 26 SEM, t_(38)_ = 6.905, p < 0.001, Figure 3A). This dependence of GO RTs and SSD length is the result of the staircase procedure that was implemented to be able to calculate SSRTs and that can correct for differences in GO reaction times between the two species. The mean SSRTs can then be calculated by subtracting the mean SSD from the mean GO RT (Logan et al., 1997). However, we did not find a significant difference in SSRTs between pigeons and humans (SSRT_pigeon_: 216 ms ± 10 SEM, SSRT_human_: 240 ms ± 10 SEM, t_(38)_ = - 1.773, p = 0.084, Figure 3A).

For the analysis of the two experimental conditions (SCD 0 and SCD 300), which are important to determine the efficiency of multi-component behavior, a repeated measures ANOVA with the within-subject factor condition (SCD 0, SCD 300) and the between-subject factor species (pigeon, human) was calculated. We found a main effect of condition (F_(1,38)_ = 188.418, p < 0.001, η_p_^2^ = 0.832, Figure 3B), indicating that overall SCD 0 reaction times (997 ms ± 38 SEM) were significantly longer than SCD 300 reaction times (834 ms ± 34 SEM). Moreover, we found a species effect (F_(1,38)_ = 25.898, p < 0.001, η_p_^2^ = 0.405, Figure 3B) indicating that pigeons showed longer reaction times in SC conditions than humans (SC_pigeon_: 1057 ms ± 31 SEM, SC_human_: 773 ms ± 31 SEM). Most importantly, however, we did not find an interaction of the factors “species” and “condition” (F_(1,38)_ = 0.020, p = 0.889, η_p_^2^ = 0.001, Figure 3B). This indicates that the relative differences between SCD 0 and SCD 300 conditions did not change significantly between species (SCD0_pigeon_: 1139 ms ± 43 SEM, SCD300_pigeon_: 975 ms ± 36 SEM; SCD0_human_: 854 ms ± 43 SEM, SCD300_human_: 693 ms ± 38 SEM). The lack of an interaction was further supported by a Bayesian statistical analysis. The Bayesian analyses to evaluate the evidence for the null hypothesis (i.e. a lack of interaction between species and condition) revealed a Bayes factor of BF_01_ = 6.2583, which provides substantial evidence for the null hypothesis (Kass and Raftery, 1995).

The processing mode of multi-component behavior is determined by calculating the slope between the mean SCD 0 and SCD 300 reaction times. Values closer to 0 indicate a serial processing strategy (more efficient), whereas values closer to −1 indicate a rather parallel processing strategy (less efficient). We compared the slope values between pigeons and humans and did not find significant differences (slope_pigeon_: −0.55 ± 0.06 SEM, slope_human_: - 0.54 ± 0.05 SEM, t_(38)_ = −0.140, p = 0.889, Figure 3C). This indicates that the mean processing strategies in the STOP-CHANGE paradigm were comparable between humans and pigeons. The lack of difference in slope values between species was supported by a Bayesian analysis. The Bayesian analyses to evaluate the evidence for the null hypothesis (i.e. a lack of species effects) revealed a Bayes factor of BF_01_ = 6.2597, which provides substantial evidence for the null hypothesis (Kass and Raftery, 1995).

### Comparison of ZENK expression between all experimental pigeon groups

**Figure 4:**
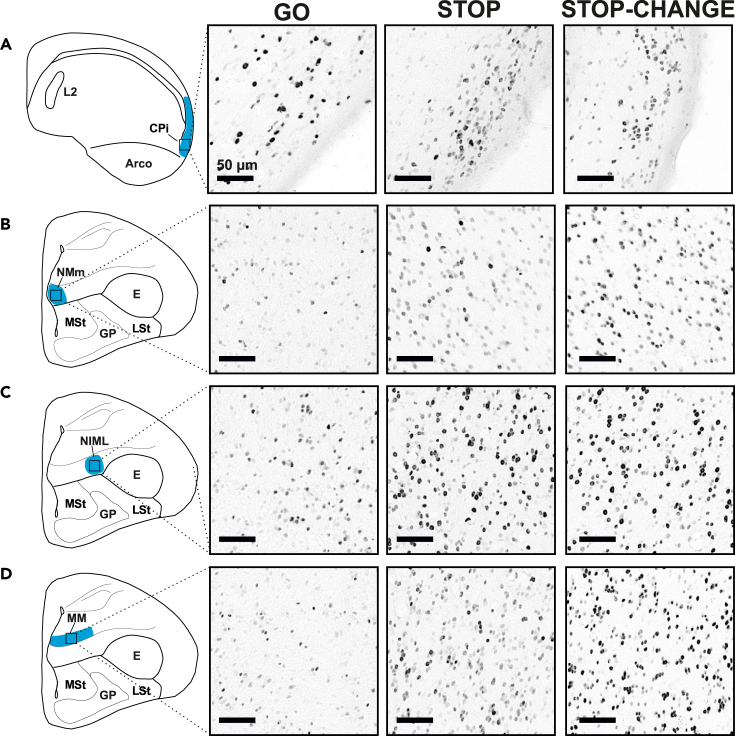
Qualitative ZENK expression in the GO, STOP and STOP-CHANGE groups in CPi, NMm, NIML and MM (A–D) Schematic illustration of the pigeon brain and microscopic images of typical ZENK expression for the GO, STOP and STOP-CHANGE groups in (A) CPi, (B) NMm, (C) NIML and (D) MM. The region of interest is highlighted in blue. All scale bars represent 50 μm. Abbrevations: *Arco: arcopallium; CPi: cortex piriformis; E: entopallium; GP: globus pallidus; LSt: lateral striatum; MM: mesopallium mediale; MSt: medial striatum, NIML: nidopallium intermedium medialis pars laterale; NMm: nidopallium mediale pars medialis.*

The number of ZENK positive cells was quantified in NMm, NIML and MM to determine their contribution to STOP-CHANGE processes/multi-component behavior. Furthermore, the cortex piriformis (CPi) was analyzed as a control area as this region belongs to the olfactory system and its activity is not expected to be modulated by STOP or STOP-CHANGE processes. Pigeons were assigned to either the GO group (n = 6), the STOP group (n = 6) or the STOP-CHANGE group (n = 8). Pigeons in the GO group were merely confronted with GO trials to control for reward and movement related neural activity in this task. Pigeons in the STOP group were confronted with GO and STOP trials to be able to dissociate STOP processes from STOP-CHANGE processes. Pigeons in the STOP-CHANGE group were the experimental group in which multi-component behavior was tested. The behavior of the STOP-CHANGE group in the final test session did not differ significantly from the pigeons of the behavioral experiment (slope_behaviorpigeons_: −0.55 ± 0.06 SEM, slope_ZENKpigeons_: −0.51 ± 0.16 SEM, t_(9.310)_ = 0.249, p = 0.809) underlining the consistency of the behavioral readout and replicability of findings. Pigeons were perfused after 400 trials had been completed and approximately 80 min after the first trial of their particular paradigm had started. This was done as the immediate-early gene ZENK has its peak activation after approximately 60 min (Mello and Ribeiro, 1998). Following immunohistochemical stainings against ZENK, neural activity was quantified in four areas of the avian telencephalon (Figure 4). Initially, ZENK expression was quantified separately for both hemispheres of all tested areas. However, as there was neither a main effect of “hemisphere” (F_(1,17)_ = 0.651, p = 0.431, η_p_^2^ = 0.037) nor an interaction between “hemisphere” and “area” (F_(3,51)_ = 0.103, p = 0.958, η_p_^2^ = 0.006), the data were pooled for further statistical analysis.

**Figure 5:**
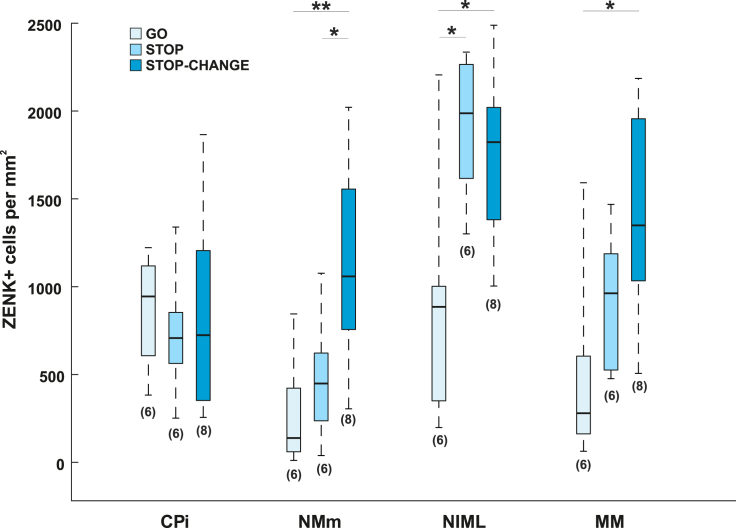
Quantitative ZENK expression in the GO, STOP and STOP-CHANGE groups in CPi, NMm, NIML and MM ZENK expression in all areas was analyzed using a repeated measures ANOVA. Bonferroni corrected pairwise comparisons revealed that ZENK expression in CPi was similar across all three groups. NMm was significantly more active in the STOP-CHANGE group compared to the STOP and to the GO groups. ZENK expression in NIML was significantly higher in the STOP-CHANGE group compared to the GO group. Furthermore, NIML was significantly more active in the STOP group compared to the GO group. ZENK expression in MM was significantly increased in the STOP-CHANGE group compared to the GO group. Numbers beneath the box plot indicate the number of tested animals. Whiskers represent the full range of data. ∗p < 0.05, ∗∗p < 0.01. Abbrevations: *CPi: cortex piriformis; MM: mesopallium mediale, NIML: nidopallium intermedium medialis pars laterale, NMm: nidopallium mediale pars medialis.*

To determine group differences in ZENK expression in all analyzed areas, a repeated measures ANOVA with the within subject factor “area” and the between subject factor “group” was calculated. We found a main effect of area (F_(3,51)_ = 22.636, p < 0.001, η_p_^2^ = 0.571) indicating that activity between analyzed areas was different. Moreover, we found a main effect of group (F_(2,17)_ = 4.744, p = 0.023, η_p_^2^ = 0.358) indicating that the GO, STOP, and STOP-CHANGE groups differed in their overall ZENK expression. Importantly, there was an interaction between the factors “area” and “group” (F_(6,51)_ = 4.805, p = 0.001, η_p_^2^ = 0.361) indicating that neural activity in the analyzed areas was different between the three conditions. Bonferroni corrected pairwise comparisons revealed that ZENK expression within the control area CPi did not differ significantly between any of the tested groups (GO group: 870 cells ±134 SEM, STOP group: 737 cells ±148 SEM, STOP-CHANGE group: 836 cells ±206 SEM, for all comparisons p > 0.999, Figures 4A and 5) indicating that differences observed in the other areas were not the result of a systematic staining artefact.

However, ZENK expression in all other areas was modulated by STOP-CHANGE processing. NMm displayed significantly more ZENK positive cells in the STOP-CHANGE group (1137 cells ± 206 SEM) compared with the GO group (273 cells ± 130 SEM, p = 0.008, Figures 4B and 5) and the STOP group (482 cells ± 145 SEM, p = 0.049, Figures 4B and 5). However, ZENK expression of the GO and the STOP group did not differ significantly (p > 0.99, Figures 4B and 5) indicating that ZENK expression in NMm is not modulated by STOP processes. For NIML, we found a different activity pattern as both the STOP-CHANGE (1739 cells ± 168 SEM, p = 0.034, Figures 4C and 5) and the STOP (1912 cells ± 161 SEM, p = 0.015, Figures 4C and 5) groups had increased ZENK expression compared with the GO group (917 cells ± 290 SEM). However, there was no significant difference in ZENK expression in NIML between the STOP and the STOP-CHANGE groups (p > 0.99, Figures 4C and 5). For MM, ZENK expression in the STOP-CHANGE group (1421 cells ± 205 SEM) was significantly higher compared to the GO group (497 cells ± 231 SEM, p = 0.015, Figures 4D and 5). However, ZENK expression in the STOP group (931 cells ± 166 SEM) did not differ significantly from the GO group (p = 0.525) or the STOP-CHANGE group (p = 0.315, Figures 4D and 5).

### Correlation of ZENK expression with the efficiency of multi-component behavior

**Figure 6:**
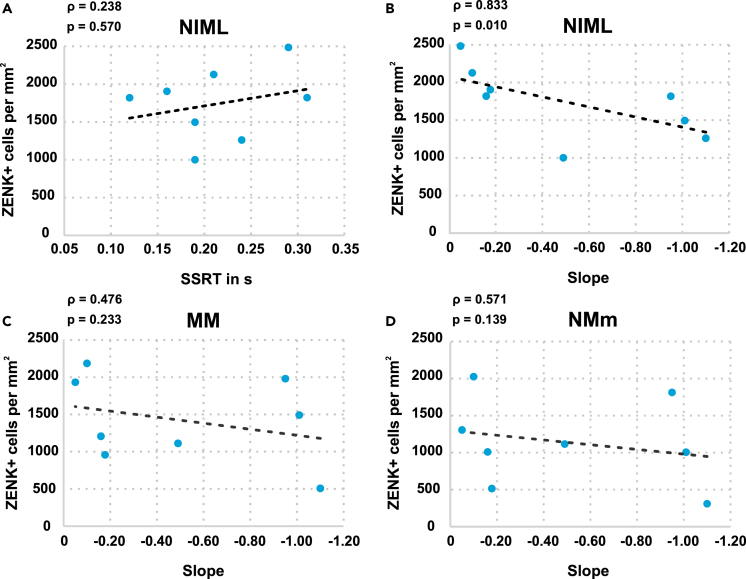
Correlation of ZENK expression with behavioral measures (A) No significant correlation of ZENK expression in NIML with the SSRTs. (B–D) Correlation of ZENK expression with the slope of the SCD-RT2 function for (B) NIML, (C) MM, (D) NMm. Increased ZENK expression is associated with a more serial processing strategy in NIML (Spearman's correlation). Abbrevations: *MM: mesopallium mediale, NIML: nidopallium intermedium medialis pars laterale, NMm: nidopallium mediale pars medialis.*

Furthermore, we investigated whether the neuronal activity of the different areas was correlated with behavioral measures of the STOP-CHANGE paradigm. As NIML showed a significant difference in ZENK expression between the GO and STOP groups, we tested whether ZENK expression in this area was correlated with SSRTs. Neither ZENK expression in NIML (ρ = 0.238, p = 0.570, Figure 6A) nor activity in the other tested areas was significantly correlated with SSRTs (MM: ρ = −0.095, p = 0.823, NMm: ρ = −0.048, p = 0.911).

In a next step, we tested whether activity in any of the investigated areas was significantly correlated with the efficiency of multi-component behavior. For this analysis, individual slope values between the CHANGE response times in the “SCD 0” and “SCD 300” condition were calculated for every pigeon of the STOP-CHANGE group (for more details see STAR Methods section). The slope value is an estimate for the processing strategy that was used during the STOP-CHANGE task. If STOP and CHANGE signals are processed in parallel, the slope values get closer to −1, whereas when STOP and CHANGE signals are processed serially, the values are closer to 0. The slope value was then correlated with the neuronal activity of all three areas. To correct for multiple comparisons and avoid alpha error accumulation the alpha significance levels were Bonferroni adjusted and set to 0.0167. We found that neuronal activity within NIML was significantly correlated with the slope of the SCD-RT2 function (ρ = 0.833, p = 0.010, Figure 6B). For the other two areas, no such correlation was found (MM: ρ = 0.476, p = 0.233, Figure 6C, NMm: ρ = 0.571, p = 0.139, Figure 6D) suggesting that there is a clear dissociation in the functional relevance of the examined brain regions for multi-component behavior.

## Discussion

In the present study, we tested pigeons and humans in highly similar versions of the STOP-CHANGE paradigm to examine how they deal with multi-component behavior situations. We found that both species are able to perform this behavior equally well. Using molecular imaging, we aimed to identify brain regions in pigeons that are most important for the examined behavioral dynamics and found that NIML within the medial nidopallium was predictive for behavioral efficiency.

To investigate the behavioral efficiency in multi-component behavior in both pigeons and humans, we employed a STOP-CHANGE paradigm where the delay between the STOP and CHANGE stimuli varied. When there is no delay, pigeons and humans have the choice to process the STOP and CHANGE task goals at the same time, or to process these goals step-by-step. However, when the delay between the STOP and CHANGE signals is longer than the time needed to respond to the STOP signal, pigeons and humans are forced to process STOP and CHANGE stimuli in a serial manner. Comparable with previous studies conducted in humans, we found that SCD 0 reaction times were significantly longer than SCD 300 reaction times in both pigeons and humans (Dippel and Beste, 2015; Mückschel et al., 2014; Stock et al., 2014). Although pigeons were overall slower than humans in both SC conditions, behavioral efficiency in the STOP-CHANGE paradigm was similar for both species. The advantage of the STOP-CHANGE paradigm for assessing behavioral efficiency is that it can control for differences in absolute reaction times between species that might occur due to different testing procedures. This is achieved by calculating the slope of the SCD-RT2 function which reflects the relationship between SCD 0 and SCD 300 reaction times (Verbruggen et al., 2008). Importantly, these slope values were highly comparable and around −0.55 for both humans and pigeons, which is in line with other studies conducted in humans (Mückschel et al., 2014; Ness and Beste 2013; Verbruggen et al., 2008). This slope value is an estimate of the efficiency of multi-component behavior and possibly indicates that pigeons and humans employ similar strategies in these behavioral situations. Therefore, it is likely that the neuronal computations underlying multi-component behavior are comparable between humans and pigeons and hence between mammals and a non-mammalian species. This is also supported by comparable SSRTs for both species which might indicate similar behavioral inhibition processes. Thus, the assessment of multi-component behavior, as done in the present study, could provide a means for cross-species comparisons in higher level cognitive functions.

Moreover, applying molecular imaging approaches, the avian brain structures most relevant for these behavioral dynamics were identified. With stainings against the immediately early gene ZENK, we found a clear functional dissociation between areas within MNM. While NMm and MM were mainly active in the STOP-CHANGE group, NIML was strongly activated in both the STOP-CHANGE and STOP groups. Crucially, only activity within NIML was correlated with the slope of the SCD-RT2 function indicating that this area is particularly relevant for the efficiency of multi-component behavior (i.e. processing strategy). Pigeons with greater NIML activity used a rather serial/more efficient processing strategy compared to pigeons with weaker activation of NIML. This finding indicates that NIML is important for the sequencing of behavior and is in line with previous studies that found that NIML is part of a system that controls the processing of sequential actions (Helduser et al., 2013; Helduser and Güntürkün, 2012). In these studies, the deactivation of NIML was associated with sequence specific errors and prolonged reaction times in serial reaction time tasks indicating that NIML monitors “what comes next” (Helduser et al., 2013; Helduser and Güntürkün, 2012). Thus far, NIML in pigeons has been mainly investigated in terms of serial order behavior, but only little else is known about the function of this area. Anatomically, NIML belongs to a pathway resembling basal ganglia thalamocortical loops in mammals, as it projects to the medial striatum which in turn sends efferents to the thalamic nucleus DLP, which projects back to NIML (Kröner and Güntürkün, 1999). This circuit has been compared to the anterior forebrain pathway in songbirds that describes the loop between the forebrain nucleus LMAN (lateral magnocellular nucleus of the nidopallium), the striatal Area X and the thalamic nucleus dorsolateralis pars medialis (DLM) (Bolhuis et al., 2010; Petkov and Jarvis, 2012). The anterior forebrain pathway is involved in song learning, song sequencing and error correction during singing (Aronov et al., 2008; Bottjer and Altenau, 2010; Doupe, 1997; Leonardo, 2004; Ölveczky et al., 2005). This suggests a functional role in hierarchical processing as song can be regarded as a form of complex and hierarchically arranged sequences of syllables (Bolhuis et al., 2010). Our data suggest that NIML is also involved in the hierarchical processing of action sequences and may thus share functional characteristics with the songbird nucleus LMAN.

Moreover, in contrast to all other areas, activity in NIML was significantly increased in the STOP group as compared with the GO group. This pattern of activation indicates that NIML is important for response inhibition which is in line with a recent fMRI study that was conducted in pigeons and found BOLD responses in NIML during a GO/NOGO paradigm (Behroozi et al., 2020). However, in our study, the activity in NIML was not significantly correlated with the SSRTs, indicating that this structure is not directly related to the efficiency of the STOP process. One explanation for the increased ZENK expression in NIML in the STOP and STOP-CHANGE groups might relate to attentional processes. In the GO group, the animals needed to perform a repetitive stimulus response task. In contrast to this, the STOP and STOP-CHANGE groups were confronted with STOP signals that could not be anticipated indicating that a change of behavior was required. Thus, NIML might be relevant for an attentional monitoring of upcoming events that is also relevant for multi-component behavior. In this regard, NIML shares functional characteristics with the rIFG, which is also implied in inhibitory control (Aron et al., 2004; Erika-Florence et al., 2014; Hampshire and Sharp, 2015) and serves overarching attentional functions that are also relevant to motor control (Hampshire et al., 2009, 2010). Moreover, the rIFG is causally involved in multi-component behavior in humans and neurophysiological changes as induced by rIFG modulations are linearly correlated with behavior (Dippel and Beste, 2015).

In contrast to NIML, ZENK expression in MM and NMm was not related to the processing mode of multi-component behavior although these two areas showed increased activity specifically in the STOP-CHANGE group. Activity in these structures might thus relate to STOP-CHANGE processing but could also reflect the planning and preparation of task relevant movements/action plans. Especially in the STOP-CHANGE group, movement preparation might be more complex as the pigeons have to give motor responses towards three target locations (GO key and two CHANGE keys) compared with one target in the GO and STOP groups (GO key). This idea is supported by a study performed in songbirds and ring doves that found activity in these areas which was correlated with the amount of limb and body movements (Feenders et al., 2008). Moreover, MNM receives multimodal input and reciprocates with NCL and the (pre)motor pallium (Kröner and Güntürkün, 1999). Thus, similar to the posterior parietal cortex in mammals in this task (Mückschel et al., 2014), NMm and MM might be involved in the transformation of sensory representations into movement plans.

### Conclusions

To conclude, the study performed a cross-species comparison of higher cognitive action functions (i.e. multi-component behavior) between humans and pigeons. We show that humans and pigeons and hence a mammalian and a non-mammalian species perform equally well in multi-component behavior and that the experimental approach used provides a vehicle for cross-species comparisons of higher-level cognitive functions. As methodological possibilities to study the neural circuits and molecular mechanisms of multitasking are limited in humans, appropriate animal models are desperately needed in this area of research to gain a mechanistic understanding and to verify existing neurocomputational models. Our study demonstrates that pigeons offer a valuable model organism based on their comparable performance to humans. Applying molecular imaging approaches in these animals, we identified brain regions that are likely most important for the examined behavioral dynamics. While NMm and MM were involved in processes specific to the STOP-CHANGE task, only NIML was relevant for the efficiency of multi-component behavior. The data suggest that NIML is particularly important for hierarchical processing during goal-directed behavior and shares functional characteristics with the human rIFG in the STOP-CHANGE paradigm. This indicates that not only the avian NCL, which is an analogous structure to the mammalian PFC (Güntürkün, 2005), but also other areas of the avian nidopallium are specialized for specific executive functions. Future studies could try to disentangle the function of NCL and NIML in multi-component behavior with causal interventions such as optogenetics, which has recently been established in pigeons (Rook et al., 2021).

### Limitations of the study

Our study aimed to investigate the role of MNM in the pigeon brain during multi-component behavior. Therefore, we screened the activity in three possible functional subdivisions within this region. For this kind of analysis, immediate-early genes offer a useful tool as the activity can be easily screened in multiple brain areas at the resolution of single cells. However, the temporal resolution of ZENK is rather low and gives an estimate of the number of cells that were active in an extended time frame (Mello and Ribeiro, 1998). Thus, with immediate-early genes, it is not possible to contrast neuronal activity that occurred during the SCD 0 condition with neuronal activity that occurred during the SCD 300 condition within the same pigeon. However, such a comparison would be interesting and insightful for the interpretation of serial and parallel processing. For example, it would be interesting to monitor activity changes in real time during the different task conditions with single cell resolution to see how cellular responses change during serial and parallel processing. These analyses are possible with electrophysiological recordings or calcium imaging and could be applied in future studies to investigate the role of NIML more thoroughly.

## STAR★Methods

### Key resources table

**Table:** 

REAGENT or RESOURCE	SOURCE	IDENTIFIER
**Antibodies**		

Normal horse serum blocking solution	Vector Laboratories, Burlingame, USA	Cat#: S-2000-20
Mouse-Anti-ZENK Antibody (7B7-A3)	Keays Lab, Vienna, Austria	Nordmann et al., 2020
Horse Anti-Mouse IgG Antibody (H+L), Biotinylated	Vector Laboratories, Burlingame, USA	Cat#: BA-2000-1.5
DAB Substrate Kit, Peroxidase (HRP), with Nickel, (3,3'-diaminobenzidine)	Vector Laboratories, Burlingame, USA	Cat#: SK-4100
VECTASTAIN® Elite ABC-HRP Kit, Peroxidase (Mouse IgG)	Vector Laboratories, Burlingame, USA	Cat#: PK-6102

**Chemicals, peptides, and recombinant proteins**		

Tissue Freezing Medium	Leica Biosystems Inc., Germany	Cat#: 14020108926
Triton™ X-100	Sigma, Steinheim, Germany	RRID: N/A

**Experimental models: organisms/strains**		

Pigeons *(Columba livia forma domestica),* wildtype	Local breeders	RRID: N/A

**Software and algorithms**		

ZEN Digital Imaging for Light Microscopy	https://www.zeiss.com/microscopy/us/products/microscope-software/zen.html#introduction	RRID:SCR_013672
ImageJ	https://imagej.net/	RRID:SCR_003070
MATLAB 2018a	https://de.mathworks.com/products/matlab.html	RRID:SCR_001622
The Biopsychology-Toolbox	Rose et al., 2008	https://sourceforge.net/projects/biopsytoolbox/
Zotero	https://www.zotero.org	RRID:SCR_013784
Presentation (Neurobehavioral Systems)	www.neurobs.com	RRID:SCR_002521
Corel Graphics Suite	https://www.coreldraw.com/	RRID:SCR_013674
IBM SPSS Statistics	https://www.ibm.com/products/spss-statistics	RRID:SCR_019096

**Other**		

ZEISS AXIO Imager.M1	Zeiss Axiocam, Jena, Germany	RRID: N/A
Microtome RM2136	Leica, Bensheim, Germany	RRID: N/A
Perfusion Pump	Ismatec, Wertheim-Modfeld, Germany	RRID: N/A

### Resource availability

#### Lead contact

Further information and requests for resources and reagents should be directed to and will be fulfilled by the lead contact, Noemi Rook (noemi.rook@rub.de).

#### Materials availability

This study did not generate new unique reagents.

### Experimental model and subject details

#### Human subjects

In this study, a total of n = 20 human subjects participated (they were aged between 21 and 25 (10 males, 10 females)). The sex of the participants had no effect on the slope of the SCD-RT2 function (t_(18)_ = 0.288, p = 0.776). All of them had normal or corrected-to-normal vision as well as normal hearing. The participants had no history of neurological or psychiatric diseases, were right-handers, and either received course credits or a financial compensation for participation in the study. This study was approved by the ethics committee of the Carl Gustav Carus Universitätsklinikum Dresden. All involved subjects provided written and informed consent before they were tested and were treated according to the Declaration of Helsinki.

#### Animal subjects

In total, n = 40 adult homing pigeons (*Columba livia*) between one and four years of age were used in this study (21 males, 19 females). The sex of the pigeon did not have an effect on the slope of the SCD-RT2 function (t_(26)_ = 0.584, p = 0.565). From these 40 pigeons, 20 pigeons were assigned to the pure behavioral experiment and were trained and tested in the STOP-CHANGE paradigm. The 20 remaining pigeons were assigned to the ZENK experiment which was further subdivided into a GO control group (n = 6), a STOP control group (n = 6) and the STOP-CHANGE group (n = 8). All pigeons were assigned to the particular groups in a randomized order. The pigeons were obtained from local breeders and were individually housed in wire-mesh cages. The housing rooms were controlled for temperature, humidity and day/night cycles (12-hour light-dark cycle). Animals were food deprived and kept at approximately 85% of their free-feeding body weight. All experiments were performed according to the principles regarding the care and use of animals adopted by the German Animal Welfare Law for the prevention of cruelty to animals as suggested by the European Communities Council Directive of November 24, 1986 (86/609/EEC) and were approved by the animal ethics committee of the Landesamt für Natur, Umwelt und Verbraucherschutz NRW, Germany. All efforts were made to minimize the number of animals used and to minimize their suffering.

### Method details

#### STOP-CHANGE paradigm for humans

The STOP-CHANGE paradigm that was used to investigate multi-component behavior in humans was thoroughly validated in previous studies (Beste et al., 2014; Mückschel et al., 2015; Verbruggen et al., 2008) and consisted of a total of 864 trials. The task was run using the Presentation software (Neurobehavioral Systems Inc.). The time between different trials was set to 900 ms and the whole paradigm was finished in approximately 25 min. The paradigm is depicted in Figure 1. All trials consisted of four vertically arranged target circles that were 8 mm in diameter and furthermore separated by three horizontal lines which were 1 mm in thickness and 8 mm in width. The reference lines and the circles were positioned 12 mm apart. The viewing angle of all stimuli was 8°. The reference lines were surrounded by a white 20 × 96 mm rectangle (line width 1 mm). Trials began with the appearance of the four potential target circle that were subdivided by the three reference lines. Following a time period of 250 ms, one of the potential white target circles was filled with white color indicating the GO stimulus. The participant's task in GO trials was to estimate whether the filled white circle was positioned below or above the reference line in the middle. The required response for the answer “above” was to press the key on the outer right using the right middle finger, while the required response for the answer “below” was to press the key on the inner right with the right index finger using a 4 key response pad. The stimuli maintained displayed till a time period of 2500 ms had expired or until the participant made their response. A normal GO trial, which occurred in 70% of the total trials was finished at that point. However, in 30% of the cases (STOP-CHANGE trials), a STOP signal was displayed. In those trials, the GO reaction needed to be suppressed and a response to a CHANGE key had to be carried out instead. Therefore, a sine tone that could be a tone of three different pitches (500 Hz, 900 Hz and 1300 Hz, all played at 74 dB) was presented via headphones. The pitch indicated which of the three lines could be considered the reference line (low tone → low reference line; middle tone → middle reference line; high tone → high reference line. The delay between the STOP and the CHANGE signal could be either 0 ms (SCD 0 condition) or 300 ms (SCD 300 condition). In those trials, the required response for the answer “above” was to press the key on the outer left using the left middle finger, while the required response for the answer “below” was to press the key on the inner left with the left index finger. The three reference lines occurred equally often in a randomized order. Furthermore, GO and STOP-CHANGE (SC) trials were mixed in a randomized fashion. Likewise, SCD 0 and SCD 300 trials, as well as the pitch of the sound was displayed in a randomized order. Thus, no preparation to any response was possible. It was stressed that the participant should react as fast as possible.

The delay between the appearance of the GO signal and the STOP signal (STOP signal delay, SSD) was adjusted by a “staircase procedure” (Verbruggen et al., 2008). This procedure was applied to end up with a probability of only 50% successful SC trials. When the participant was successful in inhibiting their GO response and later on also correctly reacted to the CHANGE key, 50 ms were added to the SSD in the next trial. When participants were unsuccessful in one of those actions, 50 ms were subtracted from the SSD in the next trial.

#### Skinner boxes

All experiments were conducted in custom-built operant chambers (33 cm (w) x 33 cm (d) x 32 cm (h)). The operant chambers were equipped with white and red LED lights and four translucent response keys (1.5 cm in diameter). One of these keys was located on the left side wall of the chamber and three were located on the rear wall. A monitor was mounted behind the rear wall of the chamber for the presentation of visual stimuli. The key on the sidewall was illuminated by a blue LED light and served as initialization key in each trial. A food hopper was affixed below the translucent response keys on the rear wall for reward presentation. Another LED was located above the food hopper that served as indicator for reward delivery and as a secondary reinforcer. The operant chamber and experimental procedure were controlled using a custom-written MATLAB code and the Biopsychology Toolbox (Rose et al., 2008).

#### STOP-CHANGE paradigm for pigeons

Pigeons were trained in a similar paradigm as humans. The paradigm is depicted in Figure 2. In pre-training, all pigeons went through an autoshaping procedure in which the birds learned to associate a pecking response on illuminated response keys with a food reward. After the pigeons successfully pecked on all four response keys (Figure 2A), they were moved to a STOP-CHANGE paradigm consisting of a grand total of 400 trials. Each trial began with a tone to signal that the blue initialization key on the sidewall was illuminated. Following a successful key peck on the initialization key, a GO stimulus (left pecking key on the rear wall) was shown after a delay of 900 ms. The delay was imposed to allow the animals to turn towards the GO stimulus. The experiment was subdivided into two trial types: in 70% of all trials, the animal was required to peck on the GO stimulus to receive a food reward (GO trial, Figure 2B). In the other 30%, a red LED STOP signal appeared after the GO stimulus that indicated that the reaction to the GO stimulus had to be inhibited. Furthermore, the animal had to change its choice from the GO stimulus to one of the two possible STOP-CHANGE (SC) stimuli that were presented next to the GO stimulus (STOP-CHANGE trial, Figures 2C and 2D). The delay between the appearance of the GO stimulus and the appearance of the STOP signal (STOP signal delay, SSD) started at 450 ms but was continuously adjusted throughout the experiment using a staircase procedure (Verbruggen et al., 2008). The staircase procedure modified the SSD so that the probability to successfully inhibit the GO response on STOP-CHANGE trials was always around 50%. To this end, the SSD was prolonged by 50 ms whenever the animal successfully inhibited the GO response to increase the likelihood of an error in the next SC trial. Likewise, an erroneous response toward the GO stimulus reduced the SSD by 50 ms decreasing the likelihood of another mistake in the next SC trial. The delay between the presentation of the STOP and the CHANGE stimulus was 0 ms in half of the SC trials (SCD 0 condition, Figure 2C) and 300 ms in the other half of the SC trials (SCD 300 condition, Figure 2D). If the pigeon pecked on the SC stimulus in an SC trial, the food hopper was raised to present a reward. In the cases where the pigeon pecked onto the GO stimulus after the STOP signal had appeared, the lights in the operant chamber were turned off for 5 s to induce a mild punishment. Individual trials were separated by a 5 s intertrial interval. The data for pigeons was collected in five consecutive sessions and pooled for further statistical analysis to achieve a high signal to noise ratio of the data.

#### GO control group (pigeons)

The GO group was the control group for all basic requirements of the task such as movement in the Skinner box, pecking and eating. This group was confronted with 100% GO trials where pecking the green stimulus was rewarded with a food access of 2 s. Apart from that the GO group received the same autoshaping procedures, was trained in the same Skinner box and was confronted with the same sensory experience.

#### STOP control group (pigeons)

The STOP group was necessary to differentiate STOP related neuronal activity from STOP-CHANGE-related neuronal activation. Pigeons in the STOP group were confronted with 70% GO trials and 30% STOP trials. In GO trials, pecking was the rewarded action, whereas in STOP trials, a red LED signaled that pecking onto the green GO key needed to be inhibited for at least 5 s. If the pigeon pecked the green stimulus incorrectly the houselight was switched off for 5 s as punishment. Apart from that, the STOP group received the same autoshaping procedures, was trained in the same Skinner box and was confronted with the same sensory experience.

#### Efficiency estimation of multi-component behavior

Psychological models propose that response selection is a process subjected to capacity limits (Mückschel et al., 2014; Stock et al., 2014; Verbruggen et al., 2008; Verbruggen and Logan, 2009). The experimental procedure employs two different SCD intervals. In the SCD 0 condition, STOP and CHANGE stimuli are presented simultaneously. Therefore, the organism can process STOP and CHANGE components of the task in parallel or serially in a step-by-step fashion. For a parallel processing approach, reaction times to the CHANGE stimulus (RT2) increase as these processes supposedly share limited capacity. If the organism processes STOP and CHANGE serially, they do not share the same limited capacity as the STOP component is already completed when the CHANGE component is initiated. Therefore, RT2s in the SCD 0 condition will be shorter if a serial strategy is employed compared to a parallel strategy. Importantly, the SCD 300 condition enforces a serial processing of the STOP- and CHANGE-related processes as the STOP component has likely finished at the time of the presentation of the CHANGE stimulus. If a serial strategy is being used in the SCD 0 condition, the RT2s between the SCD 0 and the SCD 300 should be comparable. By applying the following formula calculating the ratio of RT2 differences between the two SC conditions, one can estimate the strategy used during multi-component behavior (Verbruggen et al., 2008).

$${\text{slope}}_{\text{SCD}-\text{RT}2}\;=\frac{\text{RT}2\text{SCD}0-\text{RT}2\text{SCD}300}{\text{SCD}0-\text{SCD}300}$$

The slopes become steeper as differences between the SCD 0 and SCD 300 conditions increase. If the STOP process has not been completed when the CHANGE process is being initiated, the slope value increases indicating that multi-component behavior is more inefficient. However, if the STOP process has been completed by the time the CHANGE process is started, the slope value approaches zero indicating more efficient multi-component behavior (Verbruggen et al., 2008). Thus, a flat SCD-RT2 function reflects efficient (‘serial’) multi-component behavior processing whereas a steep slope reflects a less efficient (‘parallel’) processing strategy. Moreover, stop signal reaction times (SSRTs) were calculated by subtracting the mean SSD from the mean GO RT (Logan et al., 1997).

#### Perfusion and tissue processing

After 400 trials and approximately 80 minutes after the first trial of the paradigm had started, pigeons received intravenous injections of equithesin (0.45 ml per 100 g body weight) into the brachial vein to reduce the time variance in anesthetic uptake which can occur with intramuscular injections. This exact timing was necessary, as the immediate-early gene ZENK has its peak expression after approximately 60 minutes. The perfusion was initiated after the eyelid closure reflex was negative and the heart of the animal stopped beating. During perfusion, the pigeons were transcardially perfused with 0.9% sodium chloride (NaCl) followed by ice-cold (4°C) 4% paraformaldehyde (PFA) in 0.12 M phosphate buffer (PB; pH 7.4). After the blood had been substituted with PFA for fixation, the brain was removed from the skull and put into a postfix solution (4% PFA with 30% sucrose) at 4°C for 2 hours. Finally, the brains were cryoprotected by putting them in a 30% sucrose solution in phosphate-buffered saline (PBS; pH 7.4) for 24 hours. Prior to slicing, brains were embedded in 15% gelatin/30% sucrose and again fixated in a 4% PFA solution for 24 hours. Brains were then cut coronally in slices of 40 μm-thickness using a freezing microtome (Leica, Wetzlar, Germany).

#### ZENK immunohistochemistry

From all pigeon brains, one brain series that consisted of every tenth slice of the total brain was used for immunohistochemistry against ZENK. Sections were stained free floating and ZENK was stained permanently with a DAB (3,3 diaminobenzidine) reaction. The DAB staining was performed in accordance with the protocol of a commercially available DAB-Kit (Vector Laboratories, DAB Substrate Kit SK-4100 (Chi and Chandy, 2007)). In the beginning, sections were rinsed (3 × 10 min in PBS) and then endogenous peroxidases were blocked by incubation in 0.3% hydrogen peroxide (H_2_O_2_) in distilled water for 30 min. After further rinsing (3 × 10 min), unspecific binding sites were blocked with 30 min serum incubation (10% normal horse serum (NHS; Vector Laboratories-Vectastain Elite ABC kit) in PBS with 0.3% Triton-X-100 (PBST)). Next, slices were incubated at 4°C overnight in a monoclonal mouse anti-ZENK antibody (1:5000 in PBST, 7B7-A3). The sensitivity and selectivity of this ZENK antibody for its target has been verified with immunoblots as well as with histological stainings (Nordmann et al., 2020). On the next day, the sections were rinsed in PBS (3 × 10 min) and then incubated at room temperature for 1 hour with a secondary biotinylated anti-mouse antibody (1:1000 in PBST; Vector Laboratories-Vectastain Elite ABC kit). After further rinsing (3 × 10 min in PBS), the sections were incubated in an avidin-biotin-peroxidase complex (Vector Laboratories-Vectastain Elite ABC kit; 1:100 in PBST) for one hour. After further rinsing (3 × 10 min), the sections were transferred to the DAB solution. The solution contained 5 ml distilled water with 4 drops (100 μl) of DAB stock solution, 2 drops (84 μl) of buffer stock solution and 2 drops (80 μl) of nickel solution. The slices were transferred to cell wells and each cell well contained 1 ml of the working solution. The DAB reaction was then started by adding 6 μl H_2_O_2_ solution to each well. Following a 2 min incubation time, the sections were transferred into cell wells with PBS and rinsed (2 × 5 min in PBS). In a final step, the sections were mounted on gelatin-coated slides, dehydrated in alcohol and coverslipped with depex (Fluka, München, Germany).

### Quantification and statistical analysis

#### Quantification of ZENK activity

All slices were imaged bilaterally in 100× magnification using a ZEISS AXIO Imager.M1 with a camera (AxioCam MRm ZEISS 60N-C 2/3″0.63×). For all areas of interest ZENK positive cells were counted bilaterally. For CPi cells were counted in two consecutive slices between A 5.00 – A 6.00 and for NMm, NIML and MM cells were counted in three consecutive slices between A 9.50 – A 10.50. After that, the arithmetic mean of ZENK expression was calculated for all areas for the further statistical analysis.

#### Image analysis

Image analysis was performed as previously described (Rook et al., 2020). Intensely stained neurons within the control area CPi were taken for the upper threshold and weakly stained neurons within CPi were taken as the lower threshold. After the threshold was specified, it was kept constant for all image analyses, as staining intensities were comparable between all tested groups. Additionally, the roundness (>0.40) and the size (>15 pixel) of particles were adjusted and kept constant for all measurements. In all images the region of interest was delineated based on anatomical borders and ZENK positive cells were counted within this frame. As the investigated areas differ in their absolute size, the ZENK positive cells counted within this area were standardized to 1 mm^2^.

#### Statistical analysis

Kolmogorov-Smirnov test was used to test for the normal distribution of the data and the Levene's test to test for the homogeneity of the variance. For the behavioral analysis, a repeated measures ANOVA with the within subject factor condition (SCD 0, SCD 300) and the between subject factor species (pigeon, human) was calculated. GO RTs, SSDs, SSRTs and slope values were compared with independent samples t-tests. To correct for multiple comparisons and avoid alpha error accumulation the alpha significance values were Bonferroni adjusted and set to 0.0125. Furthermore, Bayesian statistics were calculated using MATLAB.

For the analysis of ZENK expression and to determine group differences in the number of ZENK positive cells, a repeated measures ANOVA with the within subject factors “area” and the between subject factor “group” was calculated. All post-hoc tests were Bonferroni corrected. Alpha was set to 0.05. A Spearman's correlation test was calculated to investigate the correlations between the numbers of ZENK positive cells within NIML, MM and NMm the slope of the SCD-RT2 function. To correct for multiple comparisons and avoid alpha error accumulation the alpha significance values were Bonferroni adjusted and set to 0.0167. All statistical analyses were performed with the software IBM SPSS Statistics (v. 20).

## Data Availability

• All data reported in this paper will be shared by the lead contact upon request. • This paper does not report original code. • Any additional information to reanalyze the data reported in this paper is available from the lead contact upon request.
